# Meningococcal B vaccine antigen FHbp variants among disease-causing *Neisseria meningitidis* B isolates, Italy, 2014–2017

**DOI:** 10.1371/journal.pone.0241793

**Published:** 2020-11-11

**Authors:** Anna Carannante, Cecilia Fazio, Arianna Neri, Florigio Lista, Silvia Fillo, Andrea Ciammaruconi, Paola Vacca, Paola Stefanelli

**Affiliations:** 1 Department Infectious Diseases, Istituto Superiore di Sanità, Rome, Italy; 2 Scientific Department, Army Medical Center, Rome, Italy; Uniwersytet Zielonogorski, POLAND

## Abstract

**Background:**

Typing of *Neisseria meningitidis* isolates is crucial for the surveillance of invasive meningococcal disease (IMD). We performed a molecular epidemiology study of *N*. *meningitidis* serogroup B (MenB) causing IMD in Italy between 2014 and 2017 to describe circulating strains belonging to this serogroup, with particular regards to the two factor H-binding protein (FHbp) subfamilies present in the bivalent MenB vaccine.

**Materials and methods:**

A total of 109 culture positive and 46 culture negative MenB samples were collected within the National Surveillance System (NSS) of IMD in Italy and molecularly analyzed by conventional methods.

**Results:**

Overall, 71 MenB samples showed the FHbp subfamily A and 83 the subfamily B. The subfamily variants were differently distributed by age. The most frequent variants, A05 and B231, were associated with cc213 and cc162, respectively. All MenB with the FHbp A05 variant displayed the PorA P1.22,14 and 85.7% of them the FetA F5-5. The majority of MenB with the FHbp B231 variant showed the PorA P1.22,14 (65.4%) and 84.6%, the FetA F3-6.

**Conclusion:**

MenB circulating in Italy were characterized by a remarkable association between clonal complex and FHbp variants, although a high degree of genetic diversity observed over time. A dynamic trend in clonal complexes distribution within MenB was detected. Our results stress the importance of continued meningococcal molecular surveillance to evaluate the potential vaccine coverage of the available MenB vaccines.

## Introduction

In Italy, invasive meningococcal disease (IMD), which includes septicemia, meningitis, or both, develops in 0.28 persons/100,000 population in 2018 (http://old.iss.it/mabi/). Patients with IMD may have nonspecific symptoms early in the disease, but their condition can deteriorate rapidly. Therefore, vaccination represents the optimal strategy for the prevention of IMD [1, 2]. *N*. *meningitidis* serogroup B (MenB) today represents the main serogroup circulating in several European countries [3] including Italy (http://old.iss.it/binary/mabi/cont/Interim_Report_2018_finale.pdf) given the widespread use of recommended meningococcal C vaccination in the country [4]. The factor H-binding protein (FHbp), also referred as GNA1870 (Genome-Derived Neisseria Antigen 1870) or LP2086 (lipoprotein LP2086) [5–7], included in the 4CMenB vaccine [8, 9] and in the bivalent MenB vaccine [10, 11], induces serum bactericidal antibodies. FHbp, due to its ability to specifically bind to factor H of the human complement regulatory protein and to inhibit the alternative complement pathway [7, 12], may improve the survival of *N*. *meningitidis* in human blood. Furthermore, FHbp, based on the variation of the amino acid sequence, can be divided into two subfamilies, A and B [10, 13] with the 83%-99% sequence identity within and the 60–75% between subfamilies, while by other authors FHbp is classified in three variants (v.1, v.2 and v.3) [5].

The FHbp variants included in the MenB bivalent vaccine are B01 (v1.55) and A05 (v3.45); whereas, B24 (v1.1) variant is contained in the 4CMenB vaccine [7]. Several studies are ongoing to evaluate the ability of new MenB vaccine formulations to prevent the carriage status among adolescents and adults as a major contributing factor to herd protection in a population [14, 15].

With the growing public health concern associated with meningococcal disease caused by MenB, it is important to investigate the molecular epidemiology of disease-causing *N*. *meningitidis* strains and to identify variants of FHbp from those *N*. *meningitidis* belonging to different clonal complexes.

In the present study, we investigated the two FHbp subfamilies present in the MenB bivalent vaccine. The percentage, the distribution, and the diversity patterns of FHbp variants were assessed among culture positive and culture negative MenB causing IMD in Italy from 2014 to 2017. The sequence type (ST), clonal complex (cc), PorA and FetA types and the age of the patients, were also included.

## Materials and methods

### Surveillance of invasive meningococcal disease

The National Surveillance System (NSS) for IMD is based on mandatory reporting to the Ministry of Health and to the Italian Institute of Public Health (Istituto Superiore di Sanità, ISS, http://www.iss.it/mabi). The ISS, as National Reference Laboratory (NRL) is the coordinator of the NSS, as already reported [16].

Data were analyzed using EpiInfo software (version 3.5.3, 26 January 2011).

### Ethics statement

For this study, the samples were in respect of the ethical requirement and no patient identification information was presented in the study.

### Bacterial culture and serogroup identification

Isolates were cultured on Thayer-Martin agar plates with IsoVitaleX 2% (Oxoid, Ltd.) in 5% CO_2_ atmosphere at 37°C. The serogroup was identified by slide agglutination with commercial antisera (Thermo Scientific, Waltham, Massachusetts, USA) or by multiplex PCR on bacterial DNA extracted using the QiAmp mini kit (Qiagen, Hilden, Germany) [17], from an overnight culture or directly from clinical sample.

### Whole genome sequencing

For each culture positive sample, 1 ng of DNA was used for preparing libraries following the Nextera XT DNA protocol in Illumina MiSeq platform (kit v3, 600 cycles). A first quality check of the raw sequence data was performed using FastQC [18]. Reads were trimmed using the software Sickle [19] to maintain a Q score >25, and *de novo* assembly was carried out with the ABySS software version 1.5.2 (k parameter = 63) [20]. Contigs longer than 500 bp were selected using an *ad hoc* script and kept for further analysis. The final assembly ranged from 84 to 316 (median: 209) contigs per sample (N_50_: 10,999–59,092 bp; median: 19,790 bp), covering the ca 2.2 Mb of the *N*. *meningitidis* genome.

### Multilocus sequence typing (MLST), PorA, FetA and FHbp analysis

For culture positive samples, multilocus sequence typing (MLST), PorA, FetA and FHbp typing were identified *in silico* using WGS data through PubMLST.org database (http://pubmlst.org/neisseria/).

For culture negative samples, seven housekeeping genes (*abcZ*, *adk*, *aroE*, *fumc*, *pgm*, *pdhc* and *gdh*) together with *porA*, *fetA* and *fHbp* genes were amplified referring to PCR conditions available on the PubMLST.org database or using primers and amplification parameters as already described [21]. PCR was performed using Veriti 96 well instrument (Applied Biosystem, Foster City, USA) or Mastercycler personal (Eppendorf, Hamburg, Germany). Multiple sequence and amino acid alignments were performed using Chromas version 1.15 and Clustal Omega web-site (https://www.ebi.ac.uk/Tools/msa/clustalo/).

The PubMLST.org database was used to identify allele types, the FHbp subfamilies A and B and their variants and to submit new sequences for *fHbp* gene. Moreover, the PubMLST.org database was used to submit new alleles of MLST loci and STs.

## Results

### Meningococcus of serogroup B causing invasive disease

In the period 2014–2017, 174 MenB samples were sent to ISS within the NSS, of which 155 were analyzed: 19, in fact, were unsuitable for molecular analyses due to limited sample volume or to a low DNA concentration. Among the 155, 109 were culture positive and 46 culture negative. Seventy-three were from IMD cases presenting meningitis and 33 septicaemia, 21 meningitis and septicaemia. For 28 samples, the data was not available.

### Clonal complexes

MLST were obtained for 151 samples; 134 of them belonged to a known clonal complex (cc), for 17 the cc was not assigned. The main cc_s_ identified were: cc162 (34/134; 25.4%), cc41/44 (27/134 = 20%) and cc213 (20/134; 15%) followed by cc269 (15/134; 11.2%).

The majority of MenB belonged to cc162 and reached the highest value in 2016 (n = 15; 44.1%); 8 Sequence Types (ST_s_) were identified within the cc162: ST-162, ST-9465, ST-8087, ST-10812, ST-9293, ST-5573, ST8955 and ST-12193. The ST-162 was the predominant (n = 22).

MenB of cc41/44 decreased from 40.7% (n = 11) in 2014 to 26% (n = 7) in 2017and included 15 different STs. The ST-414 (n = 5) and ST-1403 (n = 5) were the most common STs.

MenB belonging to cc213 increased from 30% (n = 6) in 2016 to 55% (n = 11) in 2017 and ST-213, ST-9197 and ST-3496 were identified.

Finally, the MenB cc269 decreased from 26.6% (n = 4) in 2014 to 13.3% (n = 2) in 2016 but increased in 2017 reaching the 33.3% (n = 5). Eleven STs were identified within the cc269 and the ST-269 (n = 3), the ST-1163 (n = 2) and the ST-8554 (n = 2) as the main STs.

### FHbp subfamilies A and B

FHbp subfamily A in 71 MenB and FHbp subfamily B in 83 MenB were identified. One sample resulted a hybrid, FHbp A/B subfamily.

**Fig 1:**
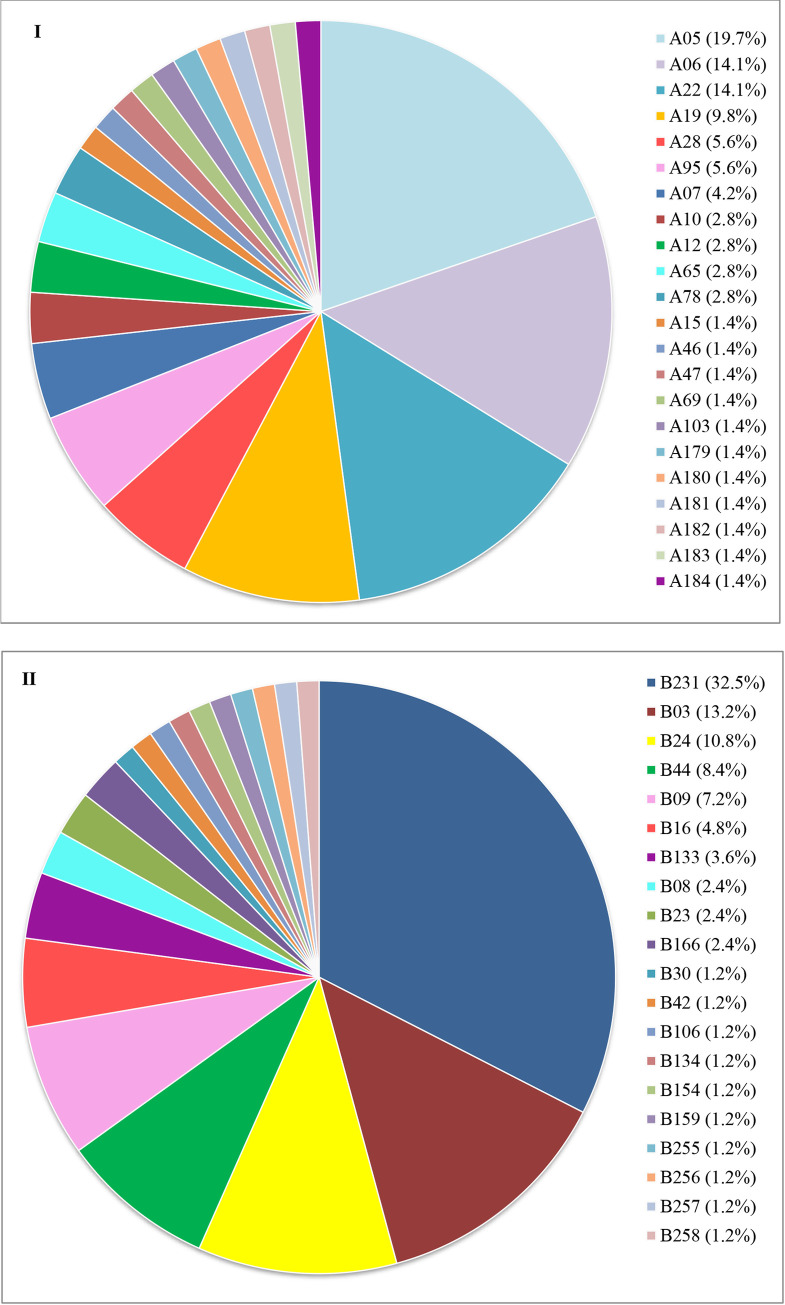
I) Distribution of variants belonging to FHbp subfamily A among 71 MenB. II) Distribution of variants belonging to FHbp subfamily B among 83 MenB.

As shown in Fig 1I, 22 variants of subfamily A were found. The A05 variant was the most frequent (n = 14; 19.7%), followed by A06 (n = 10; 14.1%) and A22 (n = 10; 14.1%).

**Fig 2:**
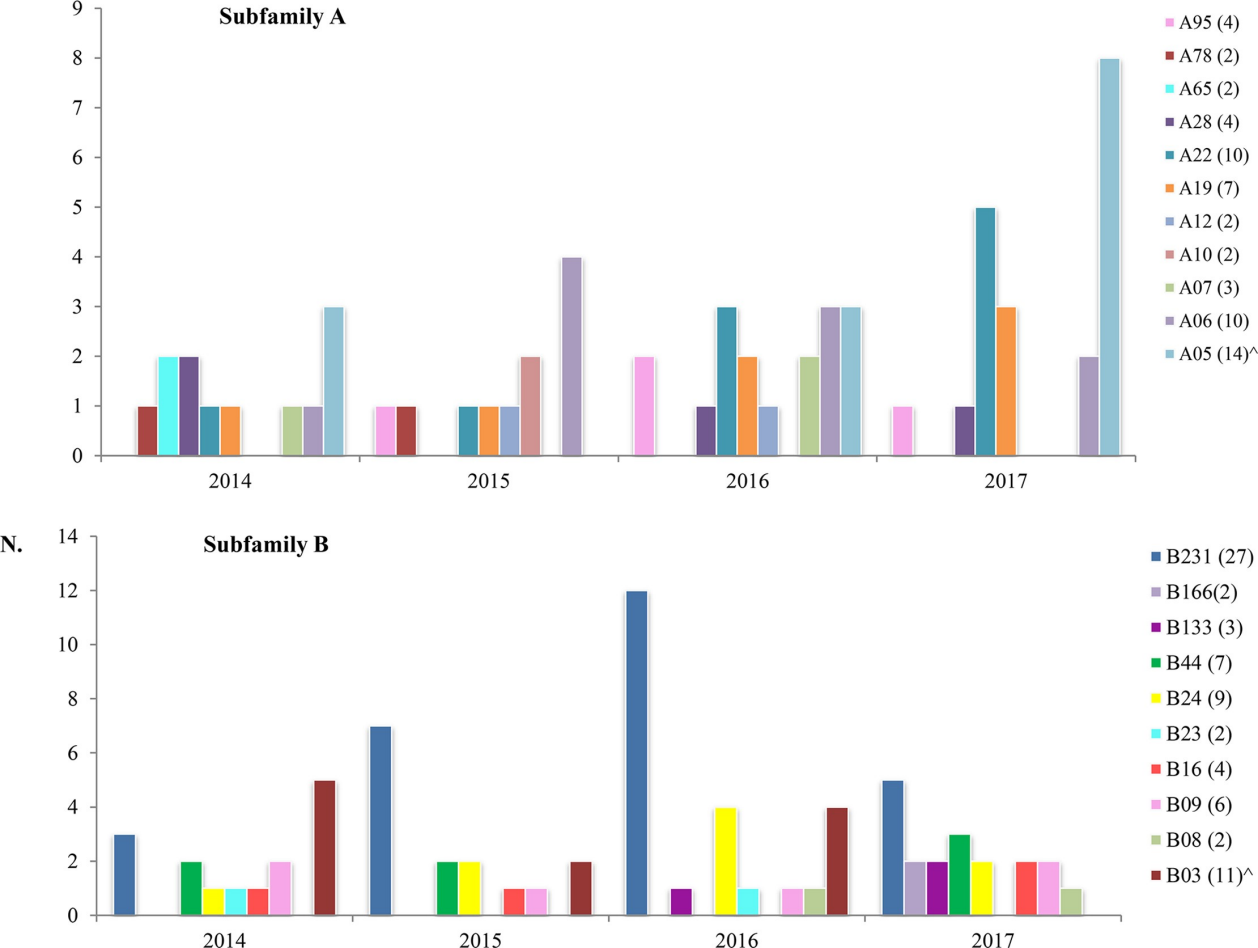
FHbp variants by year. Variants for each subfamily were included; a total of 11 for subfamily A and 10 for subfamily B. Variants represented by one sample (singleton) were not included. (^In brackets the number of culture positive and negative MenB samples).

**Fig 3:**
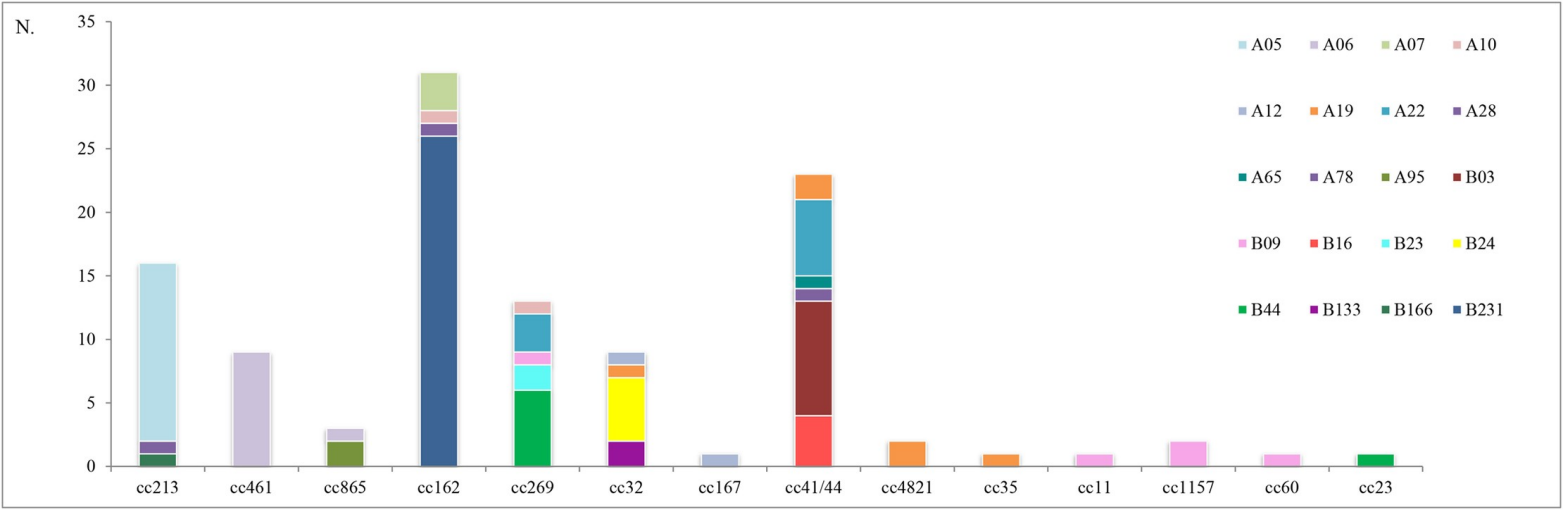
FHbp variants by clonal complexes. Variants represented by one sample (singleton) were not included.

MenB showing the A05 variant were collected from 2014 to 2017, except for 2015, and the higher number was detected in 2017 (n = 8), (Fig 2). The A05 (n = 14) was associated with cc213, (Fig 3). The A06 and A22 were identified over the 4-years of surveillance (Fig 2), most of them associated with cc461 (n = 9) and cc41/44 (n = 6), respectively (Fig 3).

Among the subfamily A, the A181, A182 and A184 were here identified and deposited in the Neisseria org web site (http://pubmlst.org/neisseria/). The A184, corresponding to *fHbp* allele 1482, presented an internal stop codon.

Fig 1II shows the 20 variants of subfamily B. The B231 (n = 27; 32.5%) was the most frequent, followed by B03 (n = 11; 13.2%) and B24 (n = 9; 10.8%).

B231 was identified through the period with a peak in 2016 (n = 12; Fig 2); all of them were associated with the cc162 (n = 26; Fig 3). One culture negative sample was not enough to obtain the cc result. The B03 variant was detected up to 2016, whilst B24 from 2014 to 2017 with the higher number in 2016 (n = 4; Fig 2). B03 and B24 variants associated mostly to cc41/44 (n = 9) and to cc32 (n = 5), respectively (Fig 3).

Within the subfamily B, FHbp variant B257 was here identified and deposited in the Neisseria org web site (http://pubmlst.org/neisseria/).

### FHbp subfamily A and B variants by age groups

**Fig 4:**
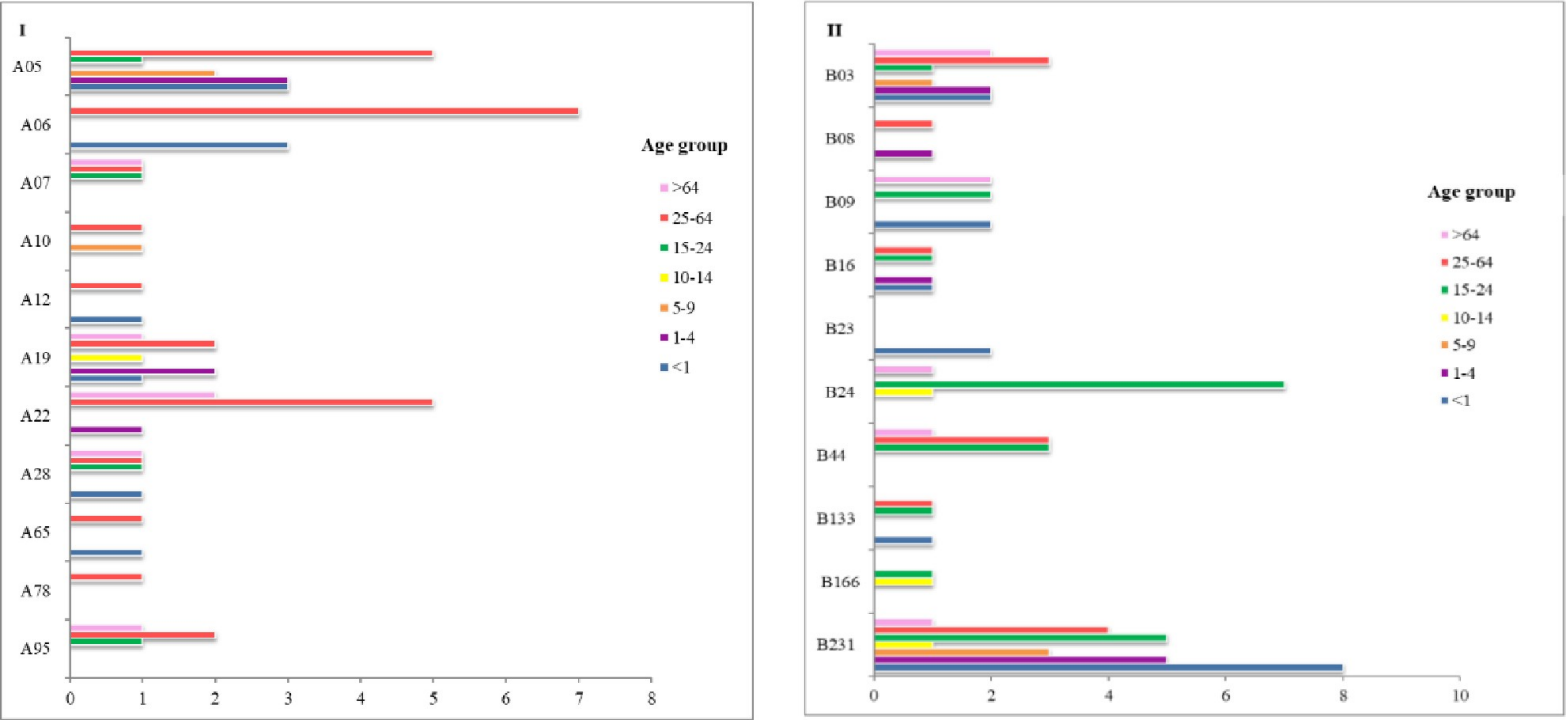
Distribution of the main FHbp subfamily A (n = 11; panel I) and B (n = 10; panel II) variants by age groups. Variants represented by one sample (singleton) were not included.

Fig 4, panel I and II, shows the distribution of the subfamily A and B variants by age groups. The age groups were those indicated in the IMD NSS report (http://www.iss.it/mabi).

Variants represented by one sample (singleton) were not included.

The majority of the subfamily A variants were detected among MenB collected from young adults and adults (age group 25‒64 years, n = 29; 40.8%), including the main variants A05 (n = 5), A06 (n = 7) and A22 (n = 5). The second large group of subfamily A variant (n = 13; 18.3%) was identified among MenB samples from infants less than 1 year of age followed by children (age group 1–4 years, n = 8; 11.3%) (Fig 4I).

The three variants, A181, A182 and A184 were found in MenB collected from patients belonged to age group15-24 years (A181) and age group <1 year (A182 and A184), (Fig 4I).

The majority of the subfamily B variants were detected among MenB collected from adolescents and young adult (age group 15‒24 years, n = 23; 27.7%). In this age group, 7 of 23 showed the B24 and 5 of 23 the B231 variants.

Additionally, B231, the most frequent, was present mainly among MenB collected from infant and children (age group 0‒9 years, n = 16; 59.2%). The remaining B231 variants were found among samples obtained from patients aged between 25–64 years (n = 4), 10–14 years (n = 1) and >64 years (n = 1), (Fig 4II).

The B03 variant was present among MenB collected in all patient age-groups, except for 10‒14 years, (Fig 4II).

### FHbp subfamily A and B variants by PorA, FetA, cc and ST

Table 1I and 1II shows the FHbp subfamily A and subfamily B by PorA (P1.VR1,VR2), FetA, cc and ST.

**Table 1:** FHbp subfamily A (I) and subfamily B (II) variants by PorA, FetA, cc and ST.

I				
Variant (n)	PorA-P1.VR1,VR2 (n)	FetA (n)	Clonal complex, cc (n)	Sequence type, ST (n)
**I**				
A05 (14)	P1.22,14 (14)	F5-5 (12)	cc213 (14)	ST-213 (9); ST-9197; ST-3496; ‒
		F3-9		ST-213
		F5-9		ST-3496
A06 (10)	P1.18–1,3 (3)/P1.18–1,13-2/P1.19–2,13–1 (3)/P1.19–2,13–2 (2)	F3-9 (9)	cc461 (9)	ST-1946 (7); ST-461; ST-7243
	P1.21,16–36	F5-8	cc865	ST-3327
A07 (3)	P1.22,14	F3-6	cc162 (3)	ST-9465
	P1.22,14/P1.7–2,4	F5-9 (2)		ST-162; ST-9293
A10 (2)	P1.22,14	F5-9	cc162	ST-8087
	P1.21–7,16	F5-36	cc269	ST-1157
A12 (2)	P1.7,16	F1-50	cc32	ST-2503
	P1.5–1,10–4	F5-36	cc167	ST-1624
A15 (1)	P1.22,26	F1-5	cc18	ST-1135
A19 (6)	P1.7–2,4 (2)	F1-5 (2)	cc41/44 (2)	ST-1403 (2)
	P1.19,15	F5-1	cc32	ST-8758
	P1.17–6,23 (2)	F3-36 (2)	cc4821 (2)	ST-3469 (2)
	P1.22–1,14	F4-1	cc35	ST-35
A22 (10)	P1.12–1,13–1	F5-5	cc41/44 (7)	ST-2719
	P1.22,9/P1.22,‒	F1-7 (2)		ST-1163; ST-5906
	P1.5,10–2 /P1.18,25/P1.18,10 /P1.18,25–10	F1-84 (4)		ST-414 (4)
	P1.22,4/P1.22,9	F5-12 (3)	cc269 (2)	ST-7610; ST-1163
	P1.22,9		‒	ST-7011
A28 (4)	P1.22,14	F5-5	cc213	ST-213
	P1.21,16 (2)	F1-14 (2)	‒ (2)	ST-11334 (2)
	P1.7–12,14	F1-7	‒	ST-1572
A46 (1)	P1.19,13–1	F3-9	cc461	ST-461
A47 (1)	P1.22,14	F5-9	cc162	ST-162
A65 (2)	P1.12–1,13	F3-6	cc41/44	ST-11336
	P1.21,16–36	F5-1	‒	ST-12740
A69 (1)	P1.22,14	F4-19	cc213	‒
A78 (2)	P1.22,‒	F3-3	cc162	ST-8087
	P1.18–24,3	F5-5	cc41/44	ST-280
A95 (3)	P1.5–3,10–2	F1-20	‒	ST-14206
	P1.7,16-36/P1.21,16–36	F5-8 (2)	cc865 (2)	ST-3327 (2)
A103 (1)	P1.18–4,25	F4-49	cc1136	ST-1136
A179 (1)	P1.18,25–10	F1-84	cc41/44	ST-414
A180 (1)	‒	‒	‒	‒
A181 (1)	P1.7–2,4	F4-1	‒	ST-11848
A182 (1)	P1.22,14	F5-5	cc213	ST-3496
A183 (1)	P1.21,16	F4-1	‒	‒
A184 (1)	P1.22,14–6	F1-5	cc41/44	ST-6349
**II**				
B03 (10)	P1.17,16–4	F2-9	cc41/44 (9)	ST-3346
	P1.17,16–4	F3-9		ST-3346
	P1.7–2,4	F5-8		ST-11851
	P1.7–2,4 (3)	F1-2 (3)		ST-1403 (3)
	P1.7–2,13-2/P1.‒,4/ ‒	F1-5 (3)		ST-11851; ST-11119; ‒
	P1.7–1,1	F5-1	‒	ST-1345
B08 (2)	P1.7–12,14/P1.‒,14	F1-7 (2)	‒ (2)	ST-1572 (2)
B09 (6)	P1.5,15	F1-5	cc60	ST-1383
	P1.21–7,16	F5-2	cc1157	ST-1157
	P1.5–1,10–8	F5-9	cc11	ST-11
	P1.22,9	F5-12 (2)	cc269	ST-1161
	‒		‒	‒
	P1.21–7,16	F5-36	cc1157	ST-1157
B16 (4)	P1.18–1,3 (2)/P1.17–1,23	F1-5 (3)	cc41/44 (4)	ST-11112; ST-4759; ST-1194
	P1.7–2,4	F4-1		ST-41
B23 (2)	P1.12–1,13-2/P1.12–1,13–7	F5-2 (2)	cc269 (2)	ST-8554 (2)
B24 (9)	P1.7,16/P1.7,30	F3-3 (3)	cc32 (2)	ST-32 (2)
	P1.‒,16		‒	‒
	P1.5–2,10/P1.19,15–23	F5-1 (4)	cc32 (2)	ST-34; ST-33
	P1.19,15 (2)		‒ (2)	ST-5151 (2)
	P1.19,15–23	F4-32	‒	‒
	P1.7,16	‒	cc32	‒
B30 (1)	P1.7,16–26	F3-3	cc32	ST-32
B42 (1)	P1.18,25–1	F5-1	cc269	ST-479
B44 (7)	P1.22,9/P1.22,14 (3)/P1.19–1,15–11	F5-1 (5)	cc269 (6)	ST-1195; ST-269 (3); ST-2693
	P1.19–1,15–11	F1-7		ST-467
	P1.17,13–1	F5-97	cc23	ST-1365
B106 (1)	P1.19–2,13–1	F3-6	cc461	ST-1946
B133 (3)	P1.18–1,30-8/P1.18–1,30–11	F3-3 (3)	cc32 (2)	ST-7460; ST-13428
	P1.10–1,30		‒	ST-32
B134 (1)	P1.18,25	F4-2	cc41/44	ST-7151
B154 (1)	P1.7–2,13–1	F1-7	‒	ST-1575
B159 (1)	P1.21,16–36	F5-8	‒	ST12464
B166 (2)	P1.22,14	F5-5	cc213	ST-213
	P1.18–17,‒	F5-69	‒	‒
B231 (26)	P1.22,14 (17)/P1.22,22/P1. ‒,14/P1.22,‒ (2)/‒	F3-6 (22)	cc162 (26)	ST-162(20); ST-10812; ‒
	P1.22,14	F3-9		‒
	P1.22,14	‒		ST-5573
	P1.‒,14/‒	‒ (2)		ST-12193; –
B255 (1)	P1.7,16	F1-106	cc32	ST-32
B256 (1)	P1.5–2,10	F5-1	‒	ST-1572
B257 (1)	P1.22,14	F3-6	cc32	ST-34
B258 (1)	P1.22,14	F5-5	cc213	ST-213

(‒ = data not available).

Within the subfamily A, all MenB (n = 14) with variant A05, displayed the PorA P1.22,14 and the majority of them the FetA F5-5 (n = 12; 85.7%), (Table 1). These samples belonged to cc213, represented by ST-213, as the predominant (n = 10; 71.4%), and by ST-9197 and ST-3496. Samples with A06 variant were mainly associated to P1.18–1,3 (n = 3) and to P1.19–2,13–1 (n = 3), the FetA F3-9 (n = 9; 90%) and cc461 (n = 9; 90%), with ST-1946 as the predominant (n = 7; 70%), (Table 1I).

The majority of MenB with the A22 variant belonged to cc41/44 (n = 7; 70%) of which ST-414 as the main ST (n = 4; 40%), (Table 1I). Several PorA and FetA alleles were found in association with A22 variant.

As shown in the Table 1I, A181 variant was found together with PorA P1.7–2,4, FetA F4-1 and ST-11848 (the cc was not assigned); A182 variant with PorA P1.22,14, FetA F5-5, cc213 and ST-3496; and A184 variant with PorA P1.22,14–6, FetA F1-5, cc41/44 and ST-6349.

The majority of MenB with the variant B231, displayed the PorA P1.22,14 (n = 17; 65.4%) and the FetA F3-6 (n = 22; 84.6%) (Table 1II) and belonged to cc162 (n = 26), with the ST-162 as the predominant ST (n = 20; 77%).

B03 variant was mainly associated with PorA P1.7–2,4 (n = 3), FetA F1-2 (n = 3) and cc41/44 (n = 9) with the ST-1403, as the predominant (Table 1II).

Finally, those showing B24 variant showed mainly PorA P1.19,15 (n = 2) or P1.19,15–23 (n = 2), associated with FetA F5-1 (n = 4) and belonged to cc32 (n = 5; 55.5%) including ST-32, ST-33 and ST-34 (Table 1II). The MenB with the new variant, B257, showed PorA P1.22,14, FetA F5-5, cc213 and ST-213 (Table 1II).

## Discussion

*Neisseria meningitidis* of serogroup B represents an major challenge in the prevention and control of invasive meningococcal disease due to this serogroup [1, 2, 4].

In this report, we provide the results of a molecular study conducted in Italy in a 4-years period, from 2014 to 2017, on the *N*. *meningitidis* strains of serogroup B causing invasive meningococcal disease and the vaccine antigen FHbp variants.

In Italy, in 2018, the incidence of MenB accounted for 0.12 cases per 100,000 inhabitants in the overall population, with a case fatality rate of 8.6%. A higher incidence of MenB was observed in the age group less than 1 year of age (1.53 cases per 100,000 inhabitants), in the age groups 10–14 (0.10 cases per 100,000 inhabitants) and 15–24 (0.25 cases per 100,000 inhabitants) years of age, data from the Italian National Surveillance System. An outbreak of MenB has described in Italy due to the switch from C to B of cc11 strain [22].

In this epidemiological situation due to its growing importance, vaccination against MenB should be considered. The National Immunization Plan (2017–2019) recommends this vaccination for infants less than one year of age and is offered free of charge [4]. In some Italian Regions, the recommendation of MenB vaccination has also been extended to other age groups (*i*. *e*. from 10 to 11 or from 11 to 12 or at 13 years of age) [23–25].

The main clonal complexes (cc_s_) characterizing the MenB collected in Italy suggest a dynamic trend in their distribution. For example, an increase of MenB cc213 and cc162 has been observed, while MenB belonging to cc41/44 decreased from 40.7% in 2014 to 26% in 2017.

Overall, all MenB harbored the *fHbp* subfamilies A or B. Seventy-one (45.8%) MenB showed the FHbp subfamily A and 83 (53.5%) the subfamily B. One sample resulted a hybrid, FHbp A/B subfamily. The distribution of the FHbp subfamilies seems to be different from what described elsewhere. Recently, in Canada [26] 63% of MenB was associated with FHbp subfamily B as well as reported in the United States, Europe, New Zealand, and South Africa [27, 28] where, combining all countries, the overall subfamily distribution was about 70% of subfamily B versus 30% subfamily A.

In this study, FHbp subfamily A was represented by 22 variants among the MenB analyzed; A05 (v3.45) variant was the most frequent (19.7%), followed by the A06 (14.1%) and A22 (14.1%). In particular, the A05 variant was the only variant identified among the meningococci belonging to cc213. As a reminder, this variant is one of two included in the bivalent MenB vaccine.

Among the 20 FHbp subfamily B variants, the main one was B231 (n = 27; 32.5%), followed by B03 (n = 11; 13.2%) and B24 (n = 9; 10.8%), also called v1.1 and included in the 4CMenB vaccine. The B231 variant was associated with meningococcal strains of cc162. In the samples collection here analyzed, B01 (v1.55), a component of the bivalent MenB vaccine, was not detected.

FHbp subfamily variants were distributed differently according to the age of the infected patients. MenB collected from young adults and adults (age group 25‒64 years) showed a high percentage of subfamily A variants (40.8%), including major variants, A05, A06 and A22. A second large group of subfamily A variants was found between MenB from infants less than one year of age (18.3%) and children 1–4 years old (11.3%). Subfamily B appears to be more distributed across all age groups with slightly different in percentage by variant. Of note, the most frequent subfamily B variant, B231, was mainly present among MenB isolated from infant and children (52.9%).

Hereby, a remarkable association has been observed between clonal complexes and FHbp variants and as previously described [27, 29–31] in some cases also with PorA allele (*e*.*g*. 100% of A05 MenB belonged to cc213 and showed PorA P1.22,14 and the 100% of B231 MenB belonged to cc162). The opposite is not always true, as neither PorA nor MLST predict the FHbp variant. Furthermore, to strengthen the association between clonal complexes and FHbp variants, B24 was always identified with cc32 and the majority of B44 with cc269, as already reported [26, 31].

The FetA variants were characterized by high antigenic diversity without any specific association with the FHbp variant.

One limitation concerns the analysis and subsequent evaluation of MenB as potentially susceptible to the MenB-FHbp vaccine as the results presented here are limited exclusively to the analysis of genetic data. In fact, the meningococcal antigen surface expression (MEASURE) assay to quantify FHbp expression, as a correlation to susceptibility to bactericidal killing [32], has not yet performed in the sample collection described. However, the presence of these variants among the Italian MenB and the sequence identity identify within the subfamilies (83%-99%) and across them (60–75%) [10, 27], is an important result to be considered in the use of the bivalent MenB vaccine.

Although relatively rare, *N*. *meningiditis* infection has long been recognized as a significant public health concern due to the high rates of morbidity and mortality associated with invasive meningococcal disease. Furthermore, outbreaks due to this pathogen are not uncommon and MenB continues to be a significant cause of disease in adolescents and young adults. Dynamic changes may occur in some variants, highlighting the need for ongoing molecular surveillance to recognize the emergence and expansion of new clones, especially those with new variants, and to investigate and possibly predict vaccine failures.

To conclude, the main findings were: *i)* a high degree of genetic diversity within serogroup B meningococci over time; *ii)* the detection of one of two subfamily FHbp variants included in the bivalent vaccine; *iii)* most of the MenB were characterized by clonal complexes common through the world and by a correlation between clonal complexes and the FHbp variant.

The study highlighted the need for continuous surveillance of circulating MenB and to molecularly characterize the genes encoding vaccine antigens, *i*.*e*. FHbp variants, to follow temporal changes in their distribution for further evaluation of vaccine composition and vaccination programs.
